# Methods for assessment of short-term coral reef fish movements within an acoustic array

**DOI:** 10.1186/2051-3933-1-7

**Published:** 2013-09-02

**Authors:** Nicholas A Farmer, Jerald S Ault, Steven G Smith, Erik C Franklin

**Affiliations:** Division of Marine Biology and Fisheries, University of Miami, Rosenstiel School for Marine and Atmospheric Science, 4600 Rickenbacker Causeway, Miami, FL 33149 USA; NOAA Fisheries, Southeast Regional Office, 263 13th Ave South, Saint Petersburg, FL 33701 USA; School of Ocean and Earth Science and Technology, Hawai’i Institute of Marine Biology, University of Hawai’i at Manoa, PO Box 1346, Kaneohe, HI 96744 USA

**Keywords:** Acoustic telemetry, Marine reserves, Acoustic array, Coral reef, Fish movements, Dry Tortugas, Reef fish

## Abstract

**Background:**

Arrays of passive receivers are a widely used tool for tracking the movements of acoustically-tagged fish in marine ecosystems; however, the spatial and temporal heterogeneity of coral reef environments pose challenges for the interpretation of tag detection data. To improve this situation for reef fishes, we introduced a novel response variable method that treats signal detections as proportions (i.e., percent transmissions detected or “detection rates”) and compared this against prior approaches to examine the influence of array and transmitter performance, signal distance and environmental factors on detection rates. We applied this method to tagged snappers and groupers in the Florida reef ecosystem and controlled range-tests on static targets in Bayboro Harbor, Florida, to provide methodological guidance for the planning and evaluation of passive array studies for coral reef fishes.

**Results:**

Logistic regression analysis indicated detection rates were primarily a non-linear function of tag distance from receiver. A ‘model-weighted’ function was developed to incorporate the non-linear relationship between detection rate and distance to provide robust positioning estimates and allow for easy extension to tags with different ping rates.

**Conclusions:**

Optimal acoustic array design requires balancing the interplay between receiver spacing, detection rates, and positioning error. Spacing receivers at twice the distance of the modeled 50% detection rate may be appropriate when quantification of overall space use is a priority, and would provide a minimum of 75% detection rate. However, for research where missing detections within the array is unacceptable or time-at-arrival based fine-scale positioning is needed, tighter receiver spacing may be required to maintain signal detection probability near 100%.

## Background

Understanding movement patterns and space use by mature fishes is critical in determining the effectiveness of marine reserves in conserving spawning stock biomass and/or providing biomass to adjacent fisheries through ‘spillover’ [1]. Recent advances in hydroacoustic monitoring technologies have made it possible to continuously and non-intrusively monitor tagged fish over long time periods [2]. It is common practice to arrange hydroacoustic receivers in overlapping arrays over broad geographic areas and apply innovative techniques to generate detailed fish movement paths that expand upon the basic presence/absence data recorded by individual receivers [1, 3–6]. Previous studies of tag detection patterns [7] have focused on mud/sand bottom environments. The spatiotemporal heterogeneity of the coral reef ecosystem may pose additional challenges for the accurate interpretation of fish detection data [8].

Over time, the number of signal receptions will be higher at nearby receivers relative to distant receivers. Assuming a linear relationship between number of detections and distance, Simpfendorfer et al. (2002) exploited this observation to compute short term centers of activity as the mean of the receiver locations weighted by the number of detections during short time batching intervals (*Δt*). Environmental heterogeneity may create variability in the relationship between probability of detection (i.e. ‘detection rate’) and distance [8–10]. Within the reef environment, each receiver in an array is exposed to a unique suite of bathymetric, geomorphological, and oceanographic conditions, possibly resulting in spatially- and temporally-distinct signal reception patterns. Additionally, detection rate at distance under fixed conditions would remain constant, but the total number of detections expected would vary for tags with different ping rates.

There is a need for practical approaches to estimating the positions of acoustically-tagged coral reef fish within spatially heterogeneous environments for tags with variable ping rates [1, 11]. Recent studies have applied time-of-arrival systems to estimate tag positions when the signal is simultaneously detected by multiple receivers [12–14]; however, the utility of these methods are limited when assessing fish home range size. Time-at-arrival positioning systems depend on either radio signals between receivers and a base station to synchronize receiver clocks (e.g., VEMCO VRAP) or reliable detections by 3–4 receivers of a stationary synchronization tag (e.g., VEMCO VPS). These requirements severely restrict receiver spacing, substantially increasing equipment costs to cover an equivalent area. When costs are not a constraint, or positioning resolution of 10 meters or less is desired within a relatively small area, use of a time-at-arrival system may be cost-appropriate. For most studies of fish home range, however, broader receiver spacing is needed to avoid undetected movements beyond the acoustic array (Farmer & Ault, *in Review*), and useful positioning estimates may be achieved by accounting for the probability of tag detection at a given distance from receiver.

In this paper, we present a method for evaluating signal detections as proportions (i.e., ‘detection rates’), and evaluate the relative influence of distance from receiver and a broad suite of environmental and bathymetric factors. Detections rates at known distances from receivers were acquired through controlled range-tests performed within arrays of hydroacoustic receivers in Bayboro Harbor, Florida and Dry Tortugas National Park, Florida. We use these calibration data to determine important considerations for data filtering and study design. Next, we extend the mean positioning methods of Simpfendorfer et al. (2002) for application in the coral reef environment by incorporating a weighted term to account for the observed non-linear decline in detection rate with distance. By incorporating detection rate, this method is also easily adjusted to account for differences in tag ping rates. Finally, we discuss balancing the interplay between receiver spacing, detection rates, positioning error, and study objectives to optimize acoustic array design.

## Results

### Calibrating receiver and tag performance

A calibration experiment performed in Bayboro Harbor, St. Petersburg, Florida (Table 1: Test 1) utilized five VR2 receivers and recorded detection rates for *n*=60 10-min time intervals for four *5s* tags tested individually and *n*=27 30-min intervals for two *120s* tags tested individually. Detection rate (*p*) was defined as the proportion of transmissions detected over a given time interval. Subsequent detections recorded by the same receiver at time intervals less than 8 seconds for *5s* tags (<1%) and less than 63 seconds for *120s* tags (<1%) were determined to be echoes and were excluded from all analyses. No differences in recorded detection rates were detected among receivers for either *5s* or *120s* tags, nor between *120s* tags (ANOVA, *p*>0.05). In contrast, recorded detection rates (detects∙10 min^*-1*^) were different for *5s* tags (ANOVA, *p* < 0.0001), and multiple comparison testing revealed small but significant differences between all four tags. Cumulatively, these differences in ping rate would have amounted to a 5-ping difference among tags when compared over 30-min intervals. Detection rates for *5s* tags decreased when deployed simultaneously (ANOVA, *p*<0.01), due to signal collisions between the two tags. Subsequent analyses incorporated these results by removing echoes, calibrating tag-specific ping rates, and testing tags individually to avoid signal interference.

**Table 1:** **Summary of acoustic receiver calibration experiments**

Test	Variables	Location	Tags	Duration	VR2	Depth_***rec***_	Method
1	Tag	Bayboro	2·*120s* 4·*5s*	90 min·*120s* ^*-1*^ 30 min·*5s* ^*-1*^	5	2.5	Tags sequentially mounted 1 m from bottom, 5.5 m from center of receiver curtain
	Receiver						
	Depth_*tag*_						
2	Tag	Tortugas II	2·*120s*	164 hrs	1	14	Tags simultaneously mounted, 2 m from bottom, 300 m from receiver
	Wind speed						
	TOD						
3	Distance	Tortugas I	1·*5s*	8 hrs	8	6 – 11	Tag suspended 3.5 m above digital video camera synced to surface GPS carried by SCUBA diver along bottom
	Rugosity						
4	Distance	Tortugas II	1·*5s*	8+ min·drop^*-1*^	14	4 – 26	Tags individually mounted, 2 m from bottom, dropped at 150 m intervals between receivers (42 drop sites)
	Depth_*rec*_						
	Depth_*tag*_						
5	Distance	Tortugas II	5·*5s* (4·*3H*, 1·*4H*)	90+ min·drop^*-1*^	12	4 – 35	Tags individually mounted, 2 m from bottom, dropped at 150 m intervals between receivers (66 drop sites)
	Wind speed						
	Depth_*rec*_						
	Tidal phase						
	Rugosity						
	Meteorological						
	Depth_*tag*_						

Where ‘VR2’ denotes number of receivers tested; ‘*120s*’ and ‘*5s*’ denotes tags with mean 120 sec and fixed 5 sec offtimes between pings, respectively; and, ‘Depth_*rec*_’ and ‘Depth_*tag*_’ denote water depth (m) at receiver site and tag drop site, respectively.

### Estimating tag distance from receiver

**Figure 1:**
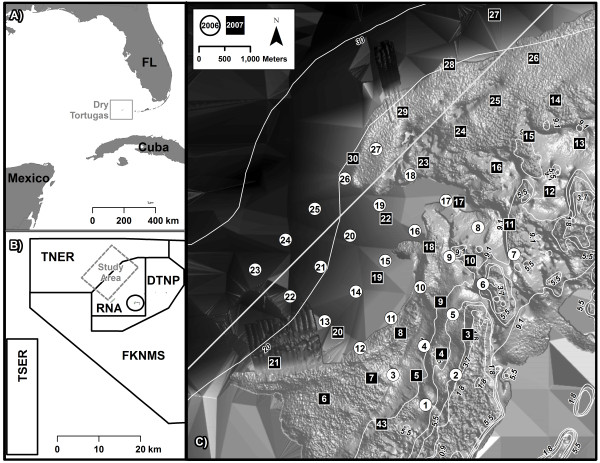
**Study area.** Maps of: **(A)** Dry Tortugas, Florida; **(B)** study site relative to management zones, including fishable (‘open’) waters of Dry Tortugas National Park (DTNP) and Florida Keys National Marine Sanctuary (FKNMS), no-take Research Natural Area (RNA), no-take Tortugas North Ecological Reserve (TNER), and no-take Tortugas South Ecological Reserve (TSER); and **(C)** hydroacoustic receiver placements in 2006 (white circles) and 2007 (black squares) overlain on bathymetry (contour lines in meters).

**Figure 2:**
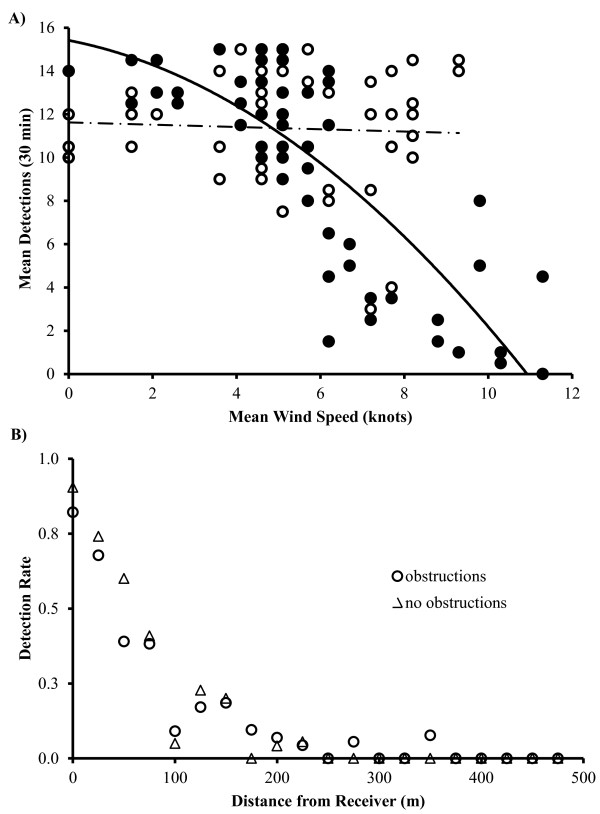
**Preliminary findings. (A)** Mean number of detections vs. mean wind speed (knots) per 30 min interval for two tags located 300 m from a receiver in Dry Tortugas National Park, FL. Differences between day (open circles, n = 51) and night (closed circles, n = 61) detection patterns are illustrated, suggesting additional ambient noise factor at night contributing to reduced detection rates at high wind speeds. **(B)** Detection rate relative to distance from receiver for a tag carried by a diver within rugose reef habitats between receivers.

Exploratory field studies in the Dry Tortugas (Figure 1) identified environmental variables in addition to tag distance from receiver that could potentially affect detection of pings (Table 1: Tests 2–5). For example, regression analysis on data from Test 2 revealed a significant declining quadratic relationship between wind speed and ‘detections∙30 min^*-1*^′ (Figure 2A; *F*_*1,129*_ = 22.1, *p* < 0.001). At night, wind speed explained 66% of the variance in detection rate, with detection rates plummeting at wind speeds above 4 knots. It is possible that the cumulative impact of biologically-induced and wind-related noise exceeded the receiver’s detection threshold. Examination of detection rates versus distance for a diver carrying a tag and a video camera (Table 1: Test 3) suggested that habitat complexity impacted detection rates within 100 m, but beyond 100 m, distance appeared to be the primary factor for missed detections (Figure 2B). Decreased detection rates were observed at increased tag distance from receivers, at shallower water depths, and in more rugose habitats (Table 1: Test 4).

A comprehensive analysis of the relationship between detection rate, distance, and environmental covariates was developed. Controlled-distance experiments recorded transmissions of *5s* tags for *n*=697 30-min time intervals over a range of distances and environmental conditions (Table 1: Test 5). Explanatory variables were tested for multicollinearity, and correlated variables were excluded from the final candidate list for inclusion in regression models (Table 2). No tag transmissions originating beyond 895 m from a receiver were detected; therefore, analyses were restricted to transmissions originating within this detection threshold. These data were used to develop two types of regression models: (1) a ’distance-only’ model relating detection rate to tag distance from receiver; and (2) a ‘full’ model relating detection rate to tag distance and environmental covariates. Estimation of the distance-only model by least-squares regression, using the number of detected pings per time interval as the response variable, was problematic due to non-normality and heteroscedasticity of residuals. These problems were not sufficiently alleviated after application of transformation and weighting procedures [15].

**Table 2:** **Candidate predictor variables investigated for inclusion in tag transmission regression models**

Predictor	Units	Description
Distance	m	Distance between tag and receiver (range 0 to 895 m)
Distance squared	m^2^	Square of distance
Tidal phase	categorical	Takes positive value at ‘slack’ tide, negative value at ‘rising/falling’ tide
Wind speed	km·h^-1^	Continuous wind speed data averaged over interval (range 0–20.9 km·h^-1^); obtained from nearby National Data Buoy Center’s ‘Pulaski Shoal Light’ C-MAN Station
Solar phase	categorical	Takes positive value during daytime, negative value at night (based on sunrise/sunset times in Tortugas)
Depth of receiver	categorical	Water depth at receiver location; takes positive value >17 m, negative value <17 m (range 4 to 34 m)

### Detection rate model

**Figure 3:**
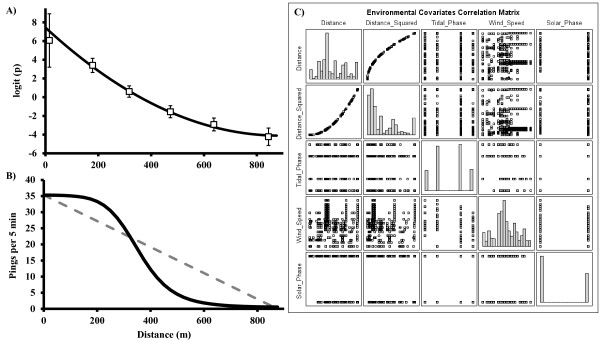
**Logistic regression analysis.** The relationship between the proportion (*p*) of tag transmissions detected by a receiver, tag distance from a receiver, and environmental covariates. **(A)** Estimates of logit(*p*) averaged over intervals of distance (open squares ± SE), and logit(*p*) modeled as a quadratic function of distance (solid line). **(B)** Predicted number of pings detected per 5-min interval from linear model (dashed line) vs. model-weighted distance-only function (solid line), illustrating systematic underestimation by linear regression (dashed line) of detections expected within 350 m of receiver and overestimation beyond 350 m. Logistic function converted to pings detected using mean tag ping rate. **(C)** Scatterplot matrix of correlations between environmental covariates. Diagonal is a histogram of values for each covariate.

**Figure 4:**
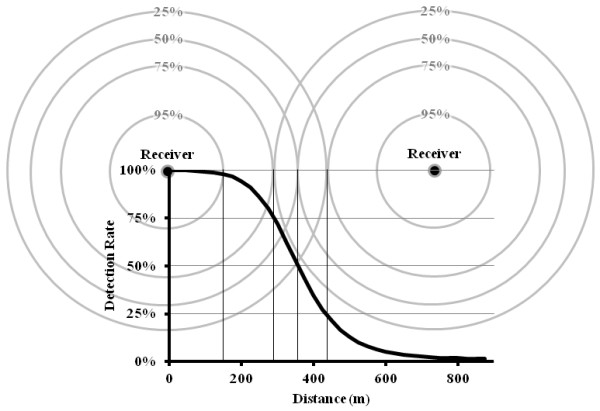
**Receiver spacing vs. probability of detection.** Application of relationship between probability of tag detection and distance to array receiver spacing, illustrating a two receiver array with overlap at 50% detection rate. Mean probability of detection distances for 25%, 75%, and 95% are also shown.

A logistic regression approach was adopted, using detection rate (*p*) as the response variable. Tag-specific ping rates determined from calibration experiments (Table 1: Test 1) were used to specify the total number of transmissions available for detection by receivers during a time interval. Estimates of logit-transformed *p* values at distance intervals of 150 m indicated a curvilinear relationship between logit(*p*) and distance (Figure 3A, open squares). A quadratic polynomial of distance provided an appropriate fit for the distance-only model (Figure 3A, solid line). Development of the full logistic regression model proceeded in a stepwise fashion, with variables added in order of importance with respect to reduction in the AICc (Table 3; [16, 17]. Chi-square likelihood ratio tests were used to determine appropriate parameters for inclusion (Tables 3 and 4). Tag distance from receiver was the most important predictor of probability of tag detection of the variables tested. The logistic model indicated that presuming a linear relationship between detection rates and receiver distance may result in systematic underestimation of the number of detections near a receiver and overestimation at further distances (Figure 3B). The relationship between distance and detection rate suggests that tight receiver spacing (<200 m) may be required in a coral reef environment to ensure 100% probability of detection, but reasonable detection rates (~>50%) may be attained with ~750 m spacing (Figure 4).

**Table 3:** **Stepwise progression of variable inclusion for logistic regression model of probability of tag detection**

			Stepwise	Cumulative
			% Reduction	% Reduction
Variable category	Predictor variable	AIC	in AIC	in AICC
Intercept	Intercept (×_0_)	963.46	–	–
Distance	Distance (×_1_)	504.84	47.60%	47.60%
Distance	Distance Squared (×_2_)	498.12	1.33%	48.30%
Water movement	Tidal phase (×_3_)	494.37	0.75%	48.69%
Ambient noise	Wind speed	ns	–	–
Ambient noise	Solar phase	ns	–	–
Depth/Rugosity	Receiver depth	ns	–	–

See Table 2 for description of explanatory variables.

**Table 4:** **Corresponding parameter estimates for the distance-only and full models (n=697 30-min time intervals)**

Parameter	Estimate	SE
**Distance-only model**		
Intercept (b_0_)	7.442	0.9947
Distance (b_1_)	-0.0261	0.0045
Distance squared (b_2_)	14.7E-06	4.58E-06
**Full model**		
Intercept (b_0_)	7.638	1.007
Distance (b_1_)	-0.0264	0.0045
Distance squared (b_2_)	14.9E-06	4.60E-06
Tidal phase^†^ (b3)	+/-0.295	0.124

See Table 2 for description of explanatory variables.

^†^ categorical variable.

The only significant environmental covariate was tidal phase (Table 4), an indicator of water current velocity, even though all the environmental variables tested influenced the probability of detection in preliminary studies. Inclusion of tidal phase provided minimal reductions in AICc [16, 17]. Correlation between environmental covariates (Table 5, Figure 3C), relatively low rates of simultaneous detections at multiple receivers, and relatively constricted ranges of values for environmental covariates may all have influenced this outcome.

**Table 5:** **Pearson correlation coefficients between environmental covariates tested for impacts on detection rate**

	Distance	Distance (squared)	Tidal phase^†^	Wind speed	Solar phase^†^	Receiver depth^†^
**Distance**	1	0.96599	-0.02025	0.04649	-0.11642	-0.01963
		*<.0001*	*0.45*	*0.0827*	*<.0001*	*0.464*
**Distance (squared)**	0.96599	1	-0.01487	0.01736	-0.05216	0.00976
	*<.0001*		*0.579*	*0.5171*	*0.0515*	*0.7158*
**Tidal phase** ^**†**^	-0.02025	-0.01487	1	-0.26643	0.15368	-0.05217
	*0.45*	*0.579*		*<.0001*	*<.0001*	*0.0515*
**Wind speed**	0.04649	0.01736	-0.26643	1	-0.13294	-0.01008
	*0.0827*	*0.5171*	*<.0001*		*<.0001*	*0.7068*
**Solar phase** ^**†**^	-0.11642	-0.05216	0.15368	-0.13294	1	-0.00556
	*<.0001*	*0.0515*	*<.0001*	*<.0001*		*0.8357*
**Receiver depth** ^**†**^	-0.01963	0.00976	-0.05217	-0.01008	-0.00556	1
	*0.464*	*0.7158*	*0.0515*	*0.7068*	*0.8357*	

Level of significance in italics.

^†^ categorical variable.

### Position estimation

**Figure 5:**
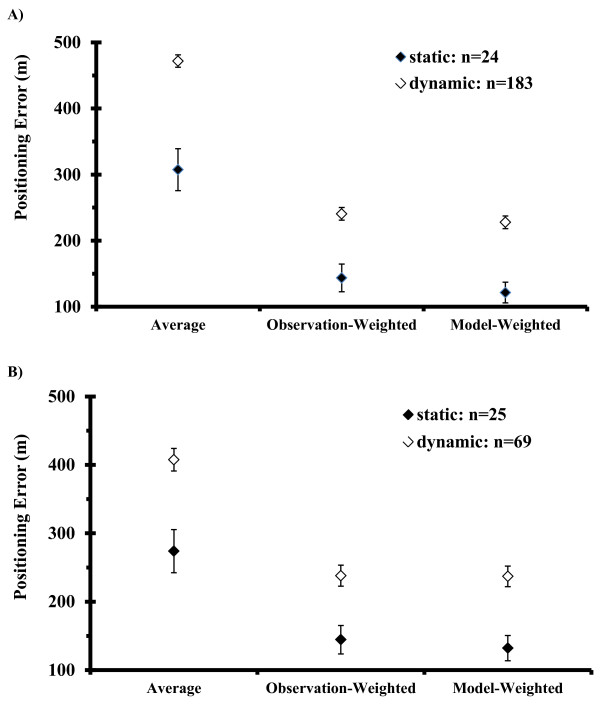
**Comparison of positioning estimators.** Illustrations of errors generated by different mean positioning estimators (±SE) for static (closed diamond) and dynamic (open diamond) **(A)** 1-min and **(B)** 5-min time intervals. The number (n) of time intervals analyzed for each experiment is denoted on the graphs.

Three time-interval batching positioning methods were tested—average, observation-weighted, and model-weighted (Table 6). For each method, the mean positioning error (*PE*) was over 100 m lower for static tags compared to dynamic tags moving higher in the water column (Figure 5). In addition, for static tests the mean *PE* decreased with an increasing number of detecting receivers (*JT Test: p*<0.0001), whereas for dynamic tests an increasing number of detecting receivers did not decrease the mean *PE* (*JT Test: p*=0.43). Estimates of mean *PE* were similar for each method for 1-min (Figure 5A) and 5-min (Figure 5B) recording intervals. Differences between harmonic and arithmetic mean position estimates were negligible; harmonic mean estimators were used for subsequent comparisons. Assuming that a tag was located at the mean spatial position of the detecting receivers (the ‘average’ method) was much less accurate than applying an observation-weighted [7] or model-weighted approach (F-test, Tukey multiple comparison test, p<0.001). Mean PEs were slightly lower for the model-weighted compared to the observation-weighted method, but these differences were not significant (p>0.05).

**Table 6:** **Formulae for calculating mean position of a tag in two dimensions**  **during a time interval** *Δ* ***t*** **for observation-weighted Simpfendorfer et al.**[7]**and model-weighted positioning estimators using arithmetic and harmonic approaches**

Method	Arithmetic		Harmonic	
Observation-weighted*				
Model-weighted				

Symbols: *n* = number of receivers in the array; *R*_*i*_ = the number of receptions at the *i* ^*th*^ receiver during time interval *Δt*; *X*_*i*_ = the X-coordinate of the *i* ^*th*^ receiver; *Y*_*i*_ = the Y-coordinate of the *i* ^*th*^ receiver; ω_*i*_ = weighting factor, ω_*i*_ = (max(*d*_*i*_)/*d*_*i*_), where *d*_*i*_ = model-estimated tag distance*, d*_*i*_=*f*(*R*_*i*_), from the *i* ^*th*^ receiver and max(*d*_*i*_) = the farthest estimated tag distance from any of the detecting receivers.

* Harmonic means corrected from Simpfendorfer et al. [7].

**Figure 6:**
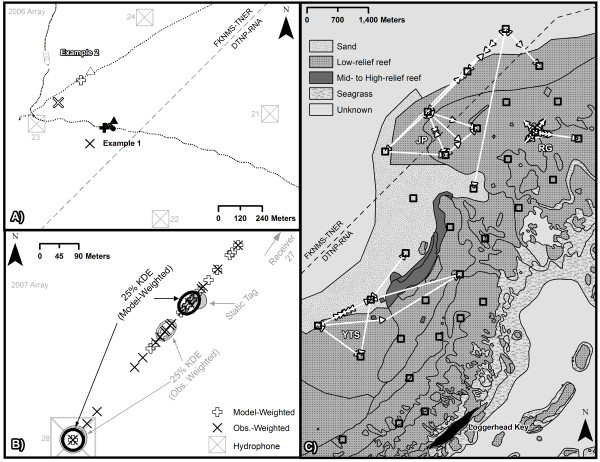
**Application of positioning estimators to data. (A)** Comparison of dynamic tag locations at 1-second intervals (circles) versus 1-minute mean position estimates generated for a single time interval using ‘average’ (triangles), ‘observation-weighted’ (X’s), and ‘model-weighted’ (crosses) methods. Examples shown are for movements between receivers (Example 1: gray and black fill) and outside the array (Example 2: white fill). **(B)** Comparison of static tag location (gray circle) versus 5-minute model-weighted and observation-weighted positioning estimates, with highest concentration of points indicated by 25% kernel density estimates (KDE; [19]; *h*=25, *c*=10). **(C)** Model-weighted 5-minute mean position estimator movement paths relative to habitat for a yellowtail snapper (YTS), jolthead porgy (JP), and red grouper (RG) inferred from receiver (black square) detection patterns. DTNP-RNA: Dry Tortugas National Park Research Natural Area, FKNMS-TNER: Florida Keys National Marine Sanctuary Tortugas North Ecological Reserve.

Plotting position estimates relative to known paths revealed that accuracy for dynamic tests was highest when movements were directly between two receivers; meanders off the fringes of the array were poorly captured by all methods (Figure 6A). For static tests, the model-weighted method provided a core of detection closer to the actual position when multiple receivers registered several detections within a batching interval (Figure 6B). Because the model-weighted time-aggregating method handles detection rate as proportions, it is easily extrapolated to longer ping rates and batching intervals. For example, Figure 6C shows tracks of several reef fish carrying VEMCO V-16 tags with different ping rates that were post-processed using this method. A 278-day track of a red grouper *Epinephelus morio* (#874; [1]) indicated that detections were registered at one core receiver and five surrounding receivers; however, nearly all detections at surrounding receivers were synchronous with detections at the core receiver. Failure to apply this method might have generated a much larger home range estimate with fringes at the surrounding receivers. The 153-day track of the yellowtail snapper *Ocyurus chrysurus* (#55; [1]) indicated oscillating movements between a few core receivers with some moderate-distance movements to surrounding receivers. The 12-day track of the jolthead porgy *Calamus bajonado* (#56; [1]) indicated some movements between many receivers followed by an exit of the array. The ability of this method to provide position fixes between receivers is well-illustrated by Figure 6C, as is the limitation of the method that detections by two receivers can only provide a position fix in line between the two receivers. Reef fish space use is commonly described using minimum convex polygon [18] or kernel density [19] home range estimation methods, both of which are sensitive to detections on the periphery of a range. By controlling for peripheral detections, the ‘model-weighted’ method avoids overestimation of space use. By providing a mean positioning error < 200 m, the ‘model-weighted’ method also allowed us to identify habitat utilization within the 200 m × 200 m categorizations identified for the Dry Tortugas region by [20]. Additional details are provided in [1].

## Discussion

Our extensive calibration work provided a few unique insights into the successful interpretation of coral reef fish movements using tag detection patterns from a passive array of acoustic receivers. First, our findings indicate the importance of data filtering for proper evaluation of acoustic signal returns. Second, using controlled range tests in two unique environments, we identified inconsistencies in tag ping rates that become an important factor in the filtering and interpretation of detection rates. Third, we presented a new method for evaluating signal detections as proportions (i.e., ‘detection rates’), and evaluated the relative influence of distance from receiver and a suite of environmental and bathymetric factors. Unlike How & de LeStang (2012), we found that the influence of environmental factors on detection rate became insignificant when considered simultaneously with distance for data collected in a broadly-spaced acoustic array. Fourth, we extended the mean positioning methods of [7] for application in the coral reef environment by incorporating a weighted term to account for the observed non-linear declines in detection rates with distance. This weighting term also handled data as proportions, meaning it is easily adjusted to account for differences in tag ping rates. Finally, we discussed balancing the interplay between receiver spacing, detection rates, positioning error, and study objectives to optimize acoustic array design. We hope that the guidance we provide in this Discussion will enhance the efficiency of future studies using passive acoustic tracking.

Calibration of receiver and tag performance suggested that data filtering is a critical element for the proper interpretation of acoustic signal returns. Our findings suggested that echoes should be eliminated from analysis, tag ping rates are variable, and signal collisions can result in failures to detect tags when deployed simultaneously. As with [21], individual receiver differences did not appear significant. When considered separately, environmental variables were important to the detection rate at fixed distances; however, logistic regression modeling indicated tag distance from receiver was by far the most important variable determining detection rate. In our coral reef study environment, the relationship between detection rate and distance was best expressed by a logistic model. A logistic ‘model-weighted’ time-aggregating positioning estimator was generated to produce useful descriptions of fish movements within home ranges and across boundaries.

By calibrating receiver and tag performance, we found that data filtering prior to evaluating reef fish movements was important to eliminate echoes caused by the reflection of the tag signal off acoustic barriers. Echoes can be determined from minimum transmission intervals for each tag. Failure to eliminate echoes could lead to overestimation of detection rate. We also found that continuous tags exhibit slight deviations from their specified ping rates which must be handled when using time-aggregating detection rates to avoid compounding minor errors and forming erroneous statistical measurements of presence-absence. Additionally, we observed signal collisions during simultaneous tag deployments, leading to failure to detect either signal [22]. Proper study design can reduce the impact of this phenomenon. A tag collision calculator is provided on the VEMCO website (http://www.vemco.com), quantifying the probability of detection of a tag given a specified ping rate and number of tags deployed simultaneously.

Preliminary field experiments revealed several variables with an impact upon detection rate. High wind speeds increased ambient noise in the water which may interfere with signal detection [10, 23, 24]. Detection rate was reduced at night, likely due to increased biological noise within the receiver bandwidth associated with snapping shrimp activity [25, 26]. Increased receiver depth reduced the signal-scattering impact of topographical features relative to available water. Habitat obstructions increased signal scattering and blockage. Increased water movement associated with tidal flow created reflective barriers, tidal bores, and eddies, increasing acoustic noise and interfering with acoustic spreading [27, 28]. Logistic regression suggested that although environmental variables may be important in fine-tuning detection rate at a given distance, the dominant explanatory variable for detection rate was a quadratic function of tag distance from receiver, with tidal phase of debatable importance as a secondary explanatory variable. Increased distance from signal origin to receiver reduced signal strength through spreading loss and absorption [22, 29]).

Our results suggested that for a spatially heterogeneous, shallow (<35 m depth) coral reef environment, a linear relationship between detection rate and distance [7] would underestimate detection rates for tags close to the receiver and overestimate detection rates for tags far from the receiver (see Figure 3B). The logistic ‘model-weighted’ function provided some advances in the process of position estimation which, when considered in aggregate, would likely provide improved estimates of fish home range utilization and movements. More substantial differences between ‘model-weighted’ and ‘observation-weighted’ positioning estimators might be expected during times when secondary variables depart substantially from their average conditions [8] or in an array with tighter receiver spacing where multiple detections at the distances of maximum deviation between the methods would be more common (e.g., Figure 3B). Regardless, the formulation of the ‘model-weighted’ estimator was advantageous for the interpretation of tag data because it appropriately handled detection data as proportions (e.g., percent transmissions detected relative to transmissions expected). This allowed for simple extrapolation of detection rates determined from rapidly-pinging tags used in receiver calibration to slower-pinging tags typically implanted in reef fish.

Selection of an appropriate time interval for batching detections is an important consideration in the development of accurate position estimates. Our experiments suggested that less movement and/or more detecting receivers during a time interval led to more accurate position estimates. Tags implanted in reef fish typically have slower ping rates relative to calibration tags [1]. Longer intervals between pings extend tag battery life and reduce signal collisions; however, they also require more time to register multiple detections. Our results suggest that a 5-min time interval might be appropriate for less mobile reef fish tagged with *120s* tags. A 5-min time interval would also reduce the impacts of false detections and signal ducting [7].

With a limited budget, optimally designing an acoustic array involves balancing the interplay between receiver spacing, detection rates, positioning error, and the particular objectives of the movement study. Our results indicated that using an estimate of maximal distance from a range-test of one receiver over a limited set of environmental conditions to determine spacing for an entire acoustic array may lead to a suboptimal array configuration, resulting in relatively low detection rates. The inflection point of the logistic function at 50% detection rate might be a more appropriate guide for receiver spacing (Figure 4). Reasonable probabilities of detection might be attained with receivers spaced at two times their 50% probability of detection. In this scenario, the combined detection rate of the tag is the union of the probability of the tag being heard by either receiver. In a two receiver array, there are four possible outcomes: (1) both receivers detect the tag, (2) neither receiver detects the tag, (3) receiver A detects the tag but receiver B does not, or (4) receiver B detects the tag but receiver A does not. Three of these scenarios result in tag detection; thus, the combined probability of detecting the tag in a two receiver array with spacing at 50% detection rate is actually 75%. As the number of receivers in the array increases, the probability of detection correspondingly increases. Unlike in a time-at-arrival system where a simultaneous detection at 3–4 receivers is needed to determine tag position at a resolution of 1–10 m, this system used in combination with the methods we have described allows for much broader spacing with a positioning resolution between 100–250 m. In our study’s coral reef environment, use of a time-at-arrival system would have required receiver spacing of around 200–250 m. For 25 receivers arranged in a 5×5 grid, this would have provided us an overall spatial coverage of 1 km^2^ with a positioning resolution of 1–10 m. Using our ‘model-weighted’ method, receiver spacing of 750–800 m was more appropriate, yielding spatial coverage of over 14 km^2^ with a positioning resolution of 100–250 m.

Broad spacing of receivers with ‘model-weighted’ positioning estimators appears appropriate when quantification of overall space use is a priority, such as developing estimates of home range size and spillover rates relative to the scale of a marine reserve [1]. In these scenarios, movements beyond the fringes of the array could introduce error that would offset improvements in positioning resolution gained from tighter spacing. However, for studies where detection gaps within the array are unacceptable, such as studies of directional movement, utilization of critical habitat features, or passage through a checkpoint, tighter receiver spacing (< 200 m) may be required to maintain probability of signal detection near 100% (Figure 4). Additionally, the percentage of time intervals with detections at multiple receivers would likely increase with tighter spacing, resulting in improved position resolution. Care should be taken to limit the number of fish being tracked simultaneously to control for signal collisions, and to tag fish near the core of the array, as passive arrays are unable to generate position estimates outside their boundaries.

Although the exact nature of the logistic relationship may vary dependent upon study location and technologies used, some preliminary calibration work following the methods we have presented may substantially improve a researcher’s ability to interpret resultant fish movement detection patterns. If funding allows, the placement of fixed position ‘calibration’ tags for the duration of the study may allow for temporally-dynamic estimation of the logistic function, reducing positioning error and reducing the need for collection of environmental information.

## Conclusions

Logistic regression analysis suggested detection rate in the coral reef environments of Dry Tortugas, Florida, was primarily a non-linear function of tag distance from receiver. Optimal acoustic array design requires balancing the interplay between receiver spacing, detection rates, positioning error, and the study objectives. Spacing receivers at twice the distance of the modeled 50% detection rate may be appropriate when quantification of overall space use is a priority. This approach provides reasonable positioning accuracy while reducing the probability of undetected movements beyond the scope of the array. However, for research where missing detections within the array is unacceptable or time-at-arrival based fine-scale positioning is needed, tighter receiver spacing may be required to maintain probability of signal detection near 100%.

## Methods

### Calibrating receiver and tag performance

The performance of hydroacoustic tags and receivers with respect to emitting and receiving pings in accordance with their respective technical specifications was evaluated in a series of controlled experiments in a homogeneous benthic environment (Table 1: Test 1). Five VEMCO VR2 (VEMCO Ltd., Nova Scotia, Canada) receivers were deployed along a line between two finger slips in Bayboro Harbor, St. Petersburg, Florida. Receivers were tested with VEMCO V16-3H (transmission strength = 158 dB re 1 μPa @ 1 m) acoustic tags. Receivers were spaced 1 m apart and suspended 1.5 m from the bottom in water 5 m deep. There were no obstructions present between receivers and transmitters, and all hydroacoustic equipment was at least 7 m from any solid object (e.g., pier or shore). Time between acoustic signal transmissions from a given tag was a function of a programmed ‘offtime’ and the time to transmit the coded pulse identifying the tag [22]. Some tags tested were configured to ping randomly every 60 – 180 sec with a mean ‘offtime’ between transmission cycles of 120 sec (‘*120s*’ tags); other tags had a fixed ‘offtime’ of 5 seconds (‘*5s*’ tags). Transmission times were approximately 3 seconds. Performance for *5s* tags was analyzed as the number of detections obtained in a 10-min interval (e.g., ‘detects∙10 min^*-1*^′). Performance for *120s* tags was analyzed as detects∙30 min^*-1*^. Tags were deployed both individually and simultaneously. Two-way analysis of variance (ANOVA) was used to test for differences in both tag and receiver performance as measured by detection rate. Analysis of data from this and subsequent experiments utilized SAS statistical software (SAS Institute, Cary, NC, USA).

### Estimating tag distance from receiver

A series of field experiments were conducted to explore the empirical relationship between the number of pings detected by a receiver and the distance of the transmitting tag from the receiver. An array of receivers was deployed in the northwestern quadrant of Dry Tortugas National Park, some 112 km west of Key West, FL, along the border of Tortugas North Ecological Reserve, covering a variety of benthic reef habitats and depths (Figure 1; [20] for detailed habitat descriptions). Spacing between receivers ranged from 600 to 1,200 m (mean = 832 m). Receivers were deployed between 4–34 m depth and mounted 5 m above the seafloor to reduce exposure to benthic noise sources, avoid signal blockage by habitat features (e.g., large blocks of coral reef), and to maintain a superior listening angle for tagged coral reef fish [30]. To reduce acoustic noise, vinyl-coated wire or 3-strand nylon line was used to attach receivers to anchors on the seafloor [31].

Experiments were conducted in two phases. The first phase entailed pilot studies in 2006, to identify environmental factors in addition to tag distance that could affect detection of pings by receivers (Table 1: Tests 2–4). These factors included wind speed, tidal phase and tidal height (proxies for water current speed), solar phase (day-night, proxy for biological noise), depth, and benthic habitat complexity/rugosity. Habitat rugosity was determined using tools from [32] applied to high-resolution bathymetric data. The second phase was a set of controlled-distance experiments in 2007, during which *5s* tags were mounted 2 m above the seafloor adjacent to a receiver, and then subsequently moved at fixed distances (usually 150 m intervals) away from the receiver (Table 1: Test 5). Tags remained at each distance interval for a minimum of 90 min. Measurements of environmental covariates identified in phase 1 studies were taken at each tag location during the experiment. Each 90+ min tag deployment was divided into 30 min intervals for analysis.

### Detection rate model

Regression models of detection rates as a function of distance and other environmental covariates were developed from the controlled-distance experiments following the general form

Two modeling approaches were evaluated: (1) least-squares regression using the number of detected pings per time interval as the response variable (*Y*) following [7]; and (2) logistic regression using detection rate (i.e., proportion of total pings detected) as the response variable. Standard model-building procedures for multiple regression were employed, including diagnostic tests for multicollinearity [33].

### Position estimation

Three methods were evaluated for estimating a tag’s position at time *t* when detected by two or more receivers during a time interval (*Δt*). The first method (termed ‘Average’) was to compute the mean position (latitude, longitude) of the detecting receivers. The second was [7] method of using the mean of receiver locations weighted by the number of detections (‘Observation-Weighted’, Table 6). The third modified the ‘Observation-Weighted’ method to incorporate regression model estimates of tag distance from a receiver (‘Model-Weighted’, Table 6). The weighting term was computed as the ratio of estimated distance between the tag and detecting receiver *i* (*d*_*i*_) relative to the farthest estimated distance of the tag from any of the detecting receivers (*d*_*max*_). The estimated distance (*d*_*i*_) is determined by inputting the observed detection rate into the logistic function. Following [7], both arithmetic and harmonic mean estimators for each method were evaluated.

Data from field experiments were used to compare the three position estimation methods under two scenarios of reef-fish movement, quiescent and actively mobile, that represented two ends of the movement spectrum. Data from the controlled-distance experiments described above were used for the quiescent or ‘static’ scenario, restricted to cases where a given tag was detected by multiple receivers during a time interval. For the actively mobile or ‘dynamic’ scenario, individual *5s* tags suspended 5m above the seafloor were towed by a slowly-moving vessel (average speed = 1.3 m∙s^-1^) through the receiver array. Position estimation methods were applied to each data series using *∆t* of 1 and 5 min. Positioning errors (*PE*) associated with each method were computed for each *Δt* as the distance between the estimated position and the actual position recorded by GPS during the experiments. To avoid potential pseudoreplication for the static experiment, the average *PE* for time intervals at the same tag location was used as a single observational unit. A computer program was written in Java 6.10 (Sun Microsystems, Inc., Santa Clara, CA) to facilitate computations of *PE*. Analysis of variance (ANOVA) was used to compare average *PE* per *∆t* among estimation methods for static and dynamic experiments. Non-parametric Jonckheere-Terpstra (JT) tests for an ordered alternative hypothesis within an independent samples (between-participants) design were used to evaluate directional trends in positioning error relative to the number of detecting receivers [34]. Positioning estimates were examined in a geographic information system using 25% kernel density estimates [19] developed with Hawth’s Tools for ArcGIS [35]. The model-weighted approach was also applied to a select subset of acoustically-tagged fish to determine its utility in creating movement paths and describing space use. Tagging methods are described in [1].

## Availability of supporting data

Supporting data are available upon request to corresponding author.
